# Induction chemotherapy in locoregionally advanced nasopharyngeal carcinoma: A systematic review and meta-analysis

**DOI:** 10.3389/fonc.2022.927510

**Published:** 2022-07-29

**Authors:** Bi-Cheng Wang, Bo-Hua Kuang, Xin-Xiu Liu, Guo-He Lin, Quentin Liu

**Affiliations:** ^1^ Cancer Center, Union Hospital, Tongji Medical College, Huazhong University of Science and Technology, Wuhan, China; ^2^ Department of Oncology, The Second Affiliated Hospital of Anhui Medical University, Hefei, China; ^3^ State Key Laboratory of Oncology in South China, Collaborative Innovation Center for Cancer Medicine, Cancer Center, Sun Yat-sen University, Guangzhou, China

**Keywords:** induction chemotherapy, nasopharyngeal carcinoma, meta-analysis, concurrent chemoradiotherapy (CCRT), responses, safety

## Abstract

**Background:**

Adding induction chemotherapy to concurrent platinum-based chemoradiotherapy has significantly prolonged the survival time of patients with locoregionally advanced nasopharyngeal carcinoma. In this study, we intend to evaluate the survival outcomes, responses, and incidences of toxicities of induction chemotherapy and the differences between different strategies.

**Methods:**

A comprehensive search was conducted in PubMed, Embase, Web of Science, and Cochrane CENTRAL on August 10, 2021. Single-arm or multi-arm prospective clinical trials on induction chemotherapy without targeted therapies or immune checkpoint inhibitors were included. Primary outcomes included survival outcomes, objective response rate, and disease control rate, and the secondary outcome was the rates of grade 3 or higher treatment-related adverse events.

**Results:**

The 39 studies included in the systematic review and meta-analysis comprised 36 clinical trials and 5389 patients. The estimates for 3-year overall and fail-free survival rates were 87% and 77%. The estimates for 5-year rates of overall and fail-free survival were 81% and 73%. Gemcitabine plus platinum and docetaxel combined with 5-fluorouracil plus platinum strategies were associated with the highest rates of 3-year and 5-year overall survival. The objective response and disease control rates were 85% and 98% after the completion of induction chemotherapy. Neutropenia (27%) and nausea/vomiting (7%) were the most common grade 3 or higher treatment-related hematological and non-hematological adverse events during the induction phase.

**Conclusions:**

Different induction chemotherapeutic strategies appear to have varying effects and risks; a comprehensive summary of the survival outcomes, responses, and toxicities in clinical trials may provide a crucial guide for clinicians.

## Introduction

It is estimated that over 70% of nasopharyngeal carcinoma (NPC) patients presented with locoregional advanced stage (1). For this population, platinum-based concurrent chemoradiotherapy (CCRT) is the backbone of the radical treatment (2, 3). For furtherly elevating the responses and prolonging survival outcomes, induction chemotherapy has been administered before CCRT. For instance, the addition of docetaxel, cisplatin, and 5-fluorouracil reduced 32% and 41% of the 3-year risks of disease progression and death (4); Gemcitabine and cisplatin induction chemotherapy significantly decreased the hazard ratio for 3-year recurrence and death by 49% and 57% in locoregionally advanced NPC patients (5). According to the latest guidelines for nasopharyngeal carcinoma, induction chemotherapy followed by CCRT is recommended as the preferred standard of care for patients with locoregionally advanced NPC (6–8).

Although adding induction chemotherapy to CCRT has been demonstrated to be superior to CCRT alone (9), substantial variations exist in different populations, induction chemotherapeutic regimens, cycles, and CCRT strategies. Ignoring these variations might lead to an inaccurate evaluation of the true efficacy and safety profile associated with induction chemotherapy.

For aiding clinical decision-making, we performed a systematic review and meta-analysis to integrate the benefits and risks of induction chemotherapy in published prospective studies and comprehensively describe the potential differences among a variety of populations, induction chemotherapeutic regimens, cycles, and CCRT strategies.

## Methods

### Search methods and study selection

We conducted this study according to the Preferred Reporting Items for Systematic Reviews and Meta-analyses (PRISMA) guideline (10). A comprehensive search of English-language prospective clinical trials was performed in PubMed, Embase, Web of Science, and Cochrane CENTRAL with the search terms (nasopharyngeal carcinoma) AND (induction chemotherapy OR neoadjuvant chemotherapy) AND (radiotherapy OR chemoradiotherapy) AND (trial OR clinical trial) on August 10, 2021. The references of relevant published clinical studies and review literatures were also searched for additional eligible trials. Inclusion criteria included: (1) Participants: over 18 years old locoregionally advanced NPC patients; (2) Interventions: induction chemotherapy followed by platinum-based CCRT; (3) Outcomes: data on survival outcomes, responses, and treatment-related adverse events were available. Single-arm and multi-arm studies were eligible. However, patients who received subsequent adjuvant chemotherapy, targeted therapy, or immunotherapy were excluded. Two authors performed the literature search and study selection independently, and any discrepancies were reviewed by a third author and resolved by consensus.

### Outcome measures and data extraction

The primary outcome measures comprised the 3- and 5-year survival rates, objective response rate (ORR, defined as the percentage of patients with a response of complete response and partial response), and disease control rate (DCR, defined as the percentage of patients with a response of complete response, partial response, and stable disease) after induction chemotherapy, at the end of CCRT, and at 3 months post CCRT. The secondary outcome was the incidence of grade 3 or higher treatment-related adverse events during induction chemotherapy and CCRT phases. Overall survival (OS) was defined as the time from diagnosis or random assignment to death because of any cause; failure-free survival (FFS) was defined as the time from diagnosis or random assignment to documented disease recurrence; locoregional recurrence-free survival (LRFS) was defined as the time from diagnosis or random assignment to locoregional disease recurrence; distant metastasis-free survival (DMFS) was defined as the time from diagnosis or random assignment to distant metastasis.

Data extraction was conducted by two authors independently and reviewed by a third author. Data regarding the number of patients, study design, region, regimens, dosing schedule, survival rates, responses, and the number of grade 3 or more adverse events were recorded.

### Statistical analysis

The response variable is the number of reported survivals, responses, and grade 3 or higher toxic effects, assumed to follow a binomial distribution. Statistical analyses were performed using R Studio (version 1.4.1717, R Foundation for Statistical Computing). The “meta” package was used to perform the random effects meta-analyses, sensitivity analyses, and tests for heterogeneity (*I* ^2^ and τ) (11). A random-effects model was selected over a fixed-effects model if *I* ^2^ > 50% because using random effects is often the preferred technique when conducting a single-arm meta-analysis to guide treatment decisions (12). τ^2^ = 0 meant that no deviations were found across the studies. Otherwise, deviations existed but did not indicate significant heterogeneity. Pooled proportions were estimated *via* the metaprop function in the “meta” package, applying a logit transformation and continuity correction of 0.5 and other default settings. The Jadad scoring scale was used to assess the quality of each eligible trial (low quality: a score of ≤ 2; high quality: a score of ≥ 3) (13). Publication bias was evaluated by funnel plots, Egger’s regression tests, and Begg’s test.

## Results

### Eligible studies and characteristics

Literature search and review of reference lists identified 1434 relevant records. After screening and eligibility assessment, we included in the meta-analysis a total of 36 prospective clinical trials involving 5389 patients (**Supplement 1**). The trials were published between 2004 and 2021, as displayed in **Table 1** (14–52). Patients in 26 trials underwent treatment in China, and patients in the other 10 trials underwent treatments in Italy, Korea, Greece, Australia, Austria, Singapore (Ethnic group: 95.3% of enrolled patients were Chinese), Switzerland, India, and Arabia. Induction chemotherapeutic regimens included (1) taxane plus platinum (TP), (2) platinum plus 5-fluorouracil (PF), (3) taxane plus platinum and 5-fluorouracil (TPF), (4) gemcitabine plus platinum (GP), (5) taxane plus platinum and epirubicin, (6) platinum plus epirubicin, (7) platinum plus capecitabine, (8) gemcitabine plus platinum and taxane, (9) mitomycin C plus epirubicin, platinum, and 5-fluorouracil, and (10) taxane plus ifosfamide and platinum. Two or three cycles of induction chemotherapy were administered. Concurrent chemoradiotherapies comprised weekly and triweekly platinum-based strategies. In addition, T3-4N0 and T3N0-1 NPC patients were excluded in Sun/Li’s and Cao/Yang’s clinical trials, respectively (32, 33, 35, 36).

**Table 1:** Characteristics of Patients and Studies.

Author	Year	Phase	Register number	Stage	No. P	Median age (range)	Male (%)	Regimens	Doses	Cycles (%)	CC	RT
Chan	2004	II	–	III-IV 5th AJCC	31	46 (31-55)	77.4	Paclitaxel Carboplatin	70 mg/m2/day, d1+8+15 AUC=6/day, d1	2 (100)	Cisplatin (40 mg/m2/wk)	2DRT
Ferrari	2008	II	–	IIb–IVb 5th AJCC	34	53 (31-57)	67.6	Cisplatin 5-Fluorouracil	100 mg/m2/day, d1 1,000 mg/m2/day, d1–4	3 (100)	Cisplatin (100 mg/m2/3wks)	3DRT
Bae	2009	II	–	III-IVb AJCC	33	Mean (SD) 50.8 (13.7)	69.7	Docetaxel Cisplatin 5-Fluorouracil	70 mg/m2/day, d1 75 mg/m2/day, d1 1,000 mg/m2/day, d1-4	3 (97.0)	Cisplatin (100 mg/m2/3wks)	–
Huang	2009	–	–	III-IV 92 Chinese stage	201	Mean (SD) 42.7 (10)	77.6	Carboplatin 5-Fluorouracil	AUC=6/day, d1 750 mg/m2/day, d1-5	2 (97.0)	Carboplatin (AUC = 6/3wks)	2DRT
Hui	2009	II	–	III-IVb 1997 UICC	34	50 (31-70)	61.8	Docetaxel Cisplatin	75 mg/m2/day, d1 75 mg/m2/day, d1	2 (100)	Cisplatin (40 mg/m2/wk)	IMRT
Kong*	2010	II	–	III-IVb 6th AJCC	59	44 (21-69)	NA	Docetaxel Cisplatin 5-Fluorouracil	75 mg/m2/day, d1 75 mg/m2/day, d1 500 mg/m2/day, d1-5	3	Cisplatin (40 mg/m2/wk)	3DRT IMRT
Zheng	2010	II	–	IIb-IVb 5th AJCC	60	48 (21-68)	71.7	Nedaplatin 5-Fluorouracil	100 mg/m2/day, d1 700 mg/m2/day, d1-4	3 (10)	Nedaplatin (100 mg/m2/3wks)	IMRT
Fountzilas	2012	II	ACTRN12609000730202	IIb-IVB 6th AJCC	72	49 (19-82)	70.8	Epirubicin Paclitaxel Cisplatin	75 mg/m2/day, d1 175 mg/m2/day, d1 75 mg/m2/day, d2	3 (86)	Cisplatin (40 mg/m2/wk)	–
Huang	2012/2015	–	–	III-IV 92 Chinese stage	201	Mean (SD) 42.7 (10)	77.6	5-Fluorouracil Carboplatin	750 mg/m2/day, d1-5 AUC=6, d1	2 (99.5)	Carboplatin (AUC = 6/3wks)	2DRT
Kong*	2013	II	NCT00816855 NCT00816816	III–IVb 6th AJCC	116	–	81	Docetaxel Cisplatin 5-Fluorouracil	75 mg/m2/day, d1 75 mg/m2/day, d1 500 mg/m2/day, d1-5	3	Cisplatin (40 mg/m2/wk)	3DRT IMRT
Lim	2013	II	–	IIb to IV 7th AJCC	28	47.4 (23-71)	67.9	Carboplatin Gemcitabine	AUC=5/day, d1 1 g/m2/day, d1, 8	3 (92.9)	Cisplatin (20 mg/m2/d1-5/3wks)	3DRT IMRT
Zhong	2013	II	–	III–IVb 6th AJCC	46	46 (22-67)	60.9	Docetaxel Cisplatin	75 mg/m2/day, d1 75 mg/m2/day, d1	2 (97.8)	Cisplatin (75 mg/m2/3wks)	–
Rosenblatt	2014	III	–	III–IV 5th UICC	139	Mean (SD) 43.5 (13.6)	74.8	Cisplatin Doxorubicin or Epirubicin or 5-Fluorouracil	100 mg/m2/day, d1 50 mg/m2/day, d1 or 75 mg/m2/day, d1 or -	2	Cisplatin (30 mg/m2/wk)	–
Lee	2015/2020	III	NCT00379262	III–IVb 6th AJCC	161	Mean (SD) 48 (9)	72	Cisplatin 5-Fluorouracil	100 mg/m2/day 1000 mg/m2//day, 120h	3	Cisplatin (100 mg/m2/3wks)	2DRT 3DRT IMRT
					165	Mean (SD) 48 (9)	80.6	Cisplatin Capecitabine	100 mg/m2/day 2000 mg/m2/14 days	3		
Tan	2015	II-III	CDR0000657121	III–IVb 97 UICC	86	48.5 (IQR 41.9-54.7)	82.6	Gemcitabine Carboplatin Paclitaxel	1000 mg/m2/day, d1+8 AUC = 2.5/day, d1+8 70 mg/m2/day, d1+8	3	Cisplatin (40 mg/m2/wk)	2DRT IMRT
Lv	2016	II	–	III–IVb 02 UICC	44	Mean (SD) 45.3 (8.4)	77.3	Docetaxel Carboplatin	70 mg/m2/day AUC=5	2 (100)	Carboplatin (AUC = 5/3wks)	–
					44	Mean (SD) 44.6 (8.9)	75	5-Fluorouracil Carboplatin	800 mg/m2/day, 4 days AUC = 5	2 (97.7)		
Sun Li	2016 2019	III	NCT01245959	III–IVb (except T3-4N0) 7th AJCC	241	42 (IQR 36-49)	80.1	Docetaxel Cisplatin 5-Fluorouracil	60 mg/m2/day, d1 60 mg/m2/day, d1 600 mg/m2/day, d1-5	3 (88)	Cisplatin (100 mg/m2/3wks)	IMRT
Tang	2016	II	NCT01479504	III-IVb 6th AJCC	113	45.05 (28-65)	78.8	Docetaxel Nedaplatin	65 mg/m2/day, d1 80 mg/m2/day, d1	2 (100)	Nedaplatin (40 mg/m2/wk)	IMRT
					110	45.32 (23-65)	77.3	Docetaxel Cisplatin	65 mg/m2/day, d1 80 mg/m2/day, d1	2 (100)	Cisplatin (40 mg/m2/wk)	
Cao Yang	2017 2019	III	NCT00705627 RDDA 2017000111	III-IVb (except T3N0-1) 6th AJCC	238	44 (19-65)	72.7	Cisplatin 5-Fluorouracil	80 mg/m2/day, d1 800 mg/m2/day, d1-5	2 (96.3)	Cisplatin (80 mg/m2/3wks)	2DRT IMRT
Ke-1	2017	II	ChiCTR-ONC-12002615	III-IVb (T3-4N0-3M0 or T1-2N2-3M0) 7th AJCC	36	48 (23-67)	77.8	Nab-paclitaxel Cisplatin	260 mg/m2/day, d1 80 mg/m2/day, d1	2 or 3	Cisplatin (80 mg/m2/3wks)	IMRT
Ke-2	2017	II	ChiCTR-ONC-12002060	III–IVb 7th AJCC	59	43 (19-59)	72.9	Lobaplatin 5-Fluorouracil	30 mg/m2/day, d1 800 mg/m2/day, d1-5	2	Lobaplatin (50 mg/m2/3wks)	IMRT
Kong*	2017	II	–	III–IVb 7th AJCC	116	–	81	Docetaxel Cisplatin 5-Fluorouracil	75 mg/m2/day, d1 75 mg/m2/day, d1 500 mg/m2/day, d1-5 every 4 weeks	3 (88.8)	Cisplatin (40 mg/m2/wk)	3DRT IMRT
Frikha	2018	III	NCT00828386 GORTEC2006-02	T2b-4N1-3	40	Mean (SD) 46 (10.2)	70	Docetaxel Cisplatin 5-Fluorouracil	75 mg/m2/day, d1 75 mg/m2/day, d1 750 mg/m2/day, d1-5	3	Cisplatin (40 mg/m2/wk)	IMRT Non-IMRT
Hong	2018	III	NCT00201396	IVa-b 5th AJCC 97 UICC	239	45 (15-69)	73.6	Mitomycin C Epirubicin Cisplatin 5-Fluorouracil	8 mg/m2/day, d1 60 mg/m2/day, d1 60 mg/m2/day, d1 450 mg/m2/day, d8	3 (84.0)	Cisplatin (30 mg/m2/wk)	3DRT IMRT
Wei	2018	CS	–	T1-4N2-3 7th AJCC	693	–	74.9	Docetaxel Cisplatin or Cisplatin 5-Fluorouracil	75 mg/m2/day, d1 75 mg/m2/day, d1 or 80 mg/m2/day, d1 1000 mg/m2/day, d1-4	2 or 4	Cisplatin (40 mg/m2/wk or 80 mg/m2/3wks)	IMRT Non-IMRT
Yang	2018	III	–	III–IVb 6th AJCC	212	- (28-70)	69.3	Paclitaxel Cisplatin or Cisplatin 5-Fluorouracil	175 mg/m2/day, d1 75 mg/m2/day, d1 or 75 mg/m2/day, d1 1000 mg/m2/day, d1-4	2 (94.8)	Cisplatin (40 mg/m2/wk)	IMRT
Ghosh-Laskar	2019	–	–	II-IVb 6th AJCC	201	42 (18-73)	72.5	Paclitaxel Ifosfamide Cisplatin or Docetaxel Cisplatin 5-Fluorouracil	175 mg/m2/day, d1 1200 mg/m2/day, d1-5 15 mg/m2/day, d2-6 or 75 mg/m2/day, d1 75 mg/m2/day, d1 750 mg/m2/day, d1-5	2 or 3	Cisplatin (30 mg/m2/wk)	IMRT
Jin	2019	NIS	NCT01536223	III-IV 7th AJCC	138	48 (18-68)	71.7	Docetaxel Cisplatin 5-Fluorouracil	75 mg/m2/day, d1 75 mg/m2/day, d1 600 mg/m2/day, d1-4	3	Cisplatin (80 mg/m2/3wks)	IMRT
					138	50 (25-69)	71	Cisplatin 5-Fluorouracil	100 mg/m2/day, d1 800 mg/m2/day, d1-5			
Lu	2019	NIS	ChiCTR-OIC-16008201	III-IVa 08 Chinese stage	60	45 (22-68)	85	Docetaxel Cisplatin 5-Fluorouracil	75 mg/m2/day, d1 75 mg/m2/day, d1 750 mg/m2/day, d1-5	2	Cisplatin (80 mg/m2/3wks)	IMRT
Zhang	2019	III	NCT01872962	III to IVb 7th AJCC	239	46 (18-64)	75.2	Gemcitabine Cisplatin	1 g/m2/day, d1+8 80 mg/m2/day, d1	3 (96.7)	Cisplatin (100 mg/m2/3wks)	IMRT
Zhao	2019	II	NCT03283293	III to IVb 6th AJCC	112	42 (14-68)	75	Cisplatin 5-Fluorouracil or Carboplatin Paclitaxel	80 mg/m2/day, d1 3.5 g/m2, d1-3 or AUC = 6, d1 135 mg/m2/day, d1	2 (100)	Cisplatin (80 mg/m2/3wks)	IMRT
Al-Rajhi	2020	II-III	NCT 03890185	III to IVb 7th AJCC	108	44 (19-70)	75.9	Docetaxel Cisplatin	75 mg/m2/day, d1 75 mg/m2/day, d1	2	Cisplatin (25 mg/m2/d1-4/wks)	IMRT
Li	2020	II	–	III to IVb 7th AJCC	58	47 (24-63)	72.4	Docetaxel Cisplatin	75 mg/m2/day, d1 80 mg/m2/day, d1	2 (89.7)	Cisplatin (80 mg/m2/3wks)	IMRT
Lv	2021	NIS III	ChiCTR-TRC-13003285	III to IVb 7th UICC	252	43.5 (36-50)	72.2	Lobaplatin 5-Fluorouracil	30 mg/m2/day, d1 800 mg/m2/day, d1-5	2	Lobaplatin (30 mg/m2/3wks)	IMRT
					250	44 (36-51)	72	Cisplatin 5-Fluorouracil	100 mg/m2/day, d1 800 mg/m2/day, d1-5	2	Cisplatin (100 mg/m2/3wks)	
Yao	2021	CS	–	III to IVb 7th AJCC/ UICC	182	–	80.2	Paclitaxel Platinum^#^ 5-Fluorouracil or Paclitaxel Platinum^#^ or Platinum^#^ 5-Fluorouracil	210 mg/m2/day, d1 40 mg/m2/day, d1-3 750 mg/m2/day, d1–3 or 210 mg/m2/day, d1 40 mg/m2/day, d1–3 or 40 mg/m2/day, d1–3 750 mg/m2/day, d1–3	1 to 4	Platinum^#^ (40 mg/m2/d1-3/3wks)	3DRT IMRT

No. P, number of patients; CC, concurrent chemotherapy; NIS, non-inferiority study; CS, cohort study; AJCC, American Joint Committee on Cancer; UICC, Union for International Cancer; 2DRT, two-dimensional radiotherapy; 3DRT, three-dimensional radiotherapy; IMRT, intensity-modulated radiotherapy; Platinum^#^, cisplatin or nedaplatin; *, included two trials.

**Supplement 2** shows the quality evaluation for each eligible study, corresponding funnel plots, Egger’s tests (P > 0.1), Begg’s test (P > 0.1), and sensitivity analyses, indicating a moderate-to-high quality for clinical trials enrolled (16 trials were identified as low quality [a score of ≤ 2], while 20 trials as high quality [a score of ≥ 3]) and the sole publication bias in the analysis of 5-year OS (Begg’s test: P = 0.09).

### Survival rates

**Figure 1:**
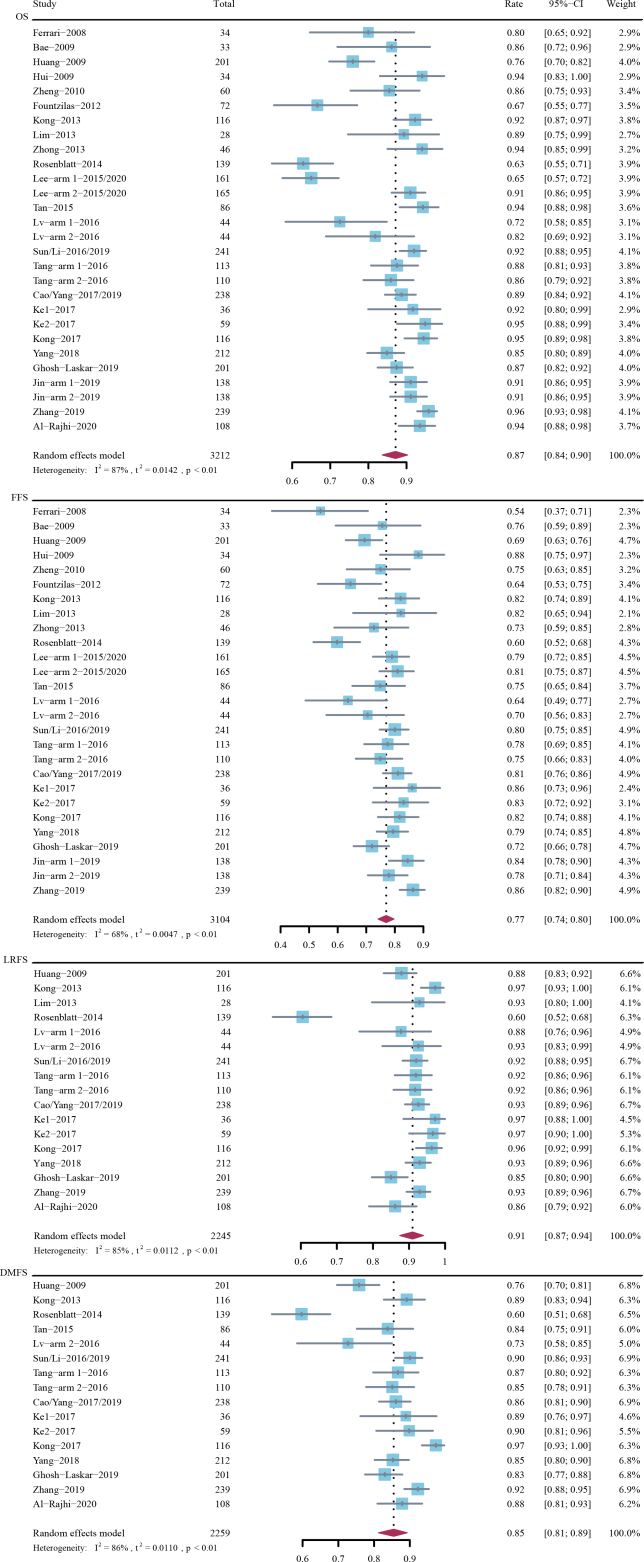
Rates of 3-year overall survival (OS), failure-free survival (FFS), locoregional recurrence-free survival (LRFS), and distant metastasis-free survival (DMFS).

The 3-year OS rate was 87% (95% CI, 84%-90%; *I* ^2^ = 87%; P < 0.01 for heterogeneity) in 3212 patients across 24 trials, the 3-year FFS rate was 77% (95% CI, 74%-80%; *I* ^2^ = 68%; P < 0.01) in 3104 patients across 24 trials, the 3-year LRFS rate was 91% (95% CI, 87%-94%; *I* ^2^ = 85%; P < 0.01) in 2245 patients across 15 trials, and the 3-year DMFS rate was 85% (95% CI, 81%-89%; *I* ^2^ = 86%; P < 0.01) in 2259 patients across 15 trials (**Figure 1**).

**Figure 2:**
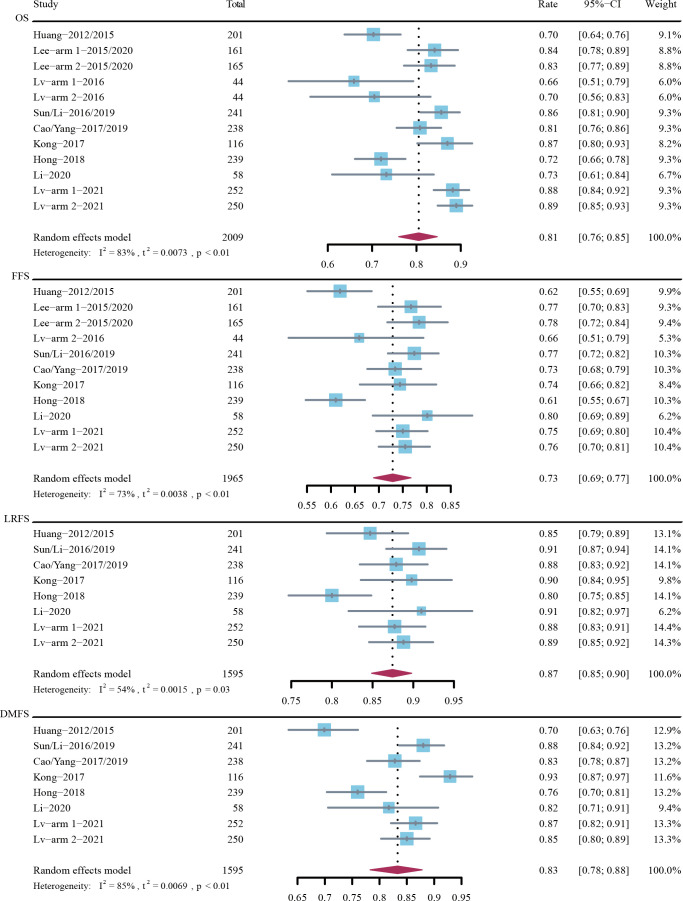
Rates of 5-year overall survival (OS), failure-free survival (FFS), locoregional recurrence-free survival (LRFS), and distant metastasis-free survival (DMFS).

The 5-year OS rate was 81% (95% CI, 76%-85%; *I* ^2^ = 83%; P < 0.01) in 2009 patients across 9 trials, the 5-year FFS rate was 73% (95% CI, 69%-77%; *I* ^2^ = 73%; P < 0.01) in 1965 patients across 9 trials, the 5-year LRFS rate was 87% (95% CI, 85%-90%; *I* ^2^ = 54%; P = 0.03) in 1595 patients across 7 trials, and the 5-year DMFS rate was 83% (95% CI, 78%-88%; *I* ^2^ = 85%; P < 0.01) in 1595 patients across 7 trials (**Figure 2**).

### Response rates

**Figure 3:**
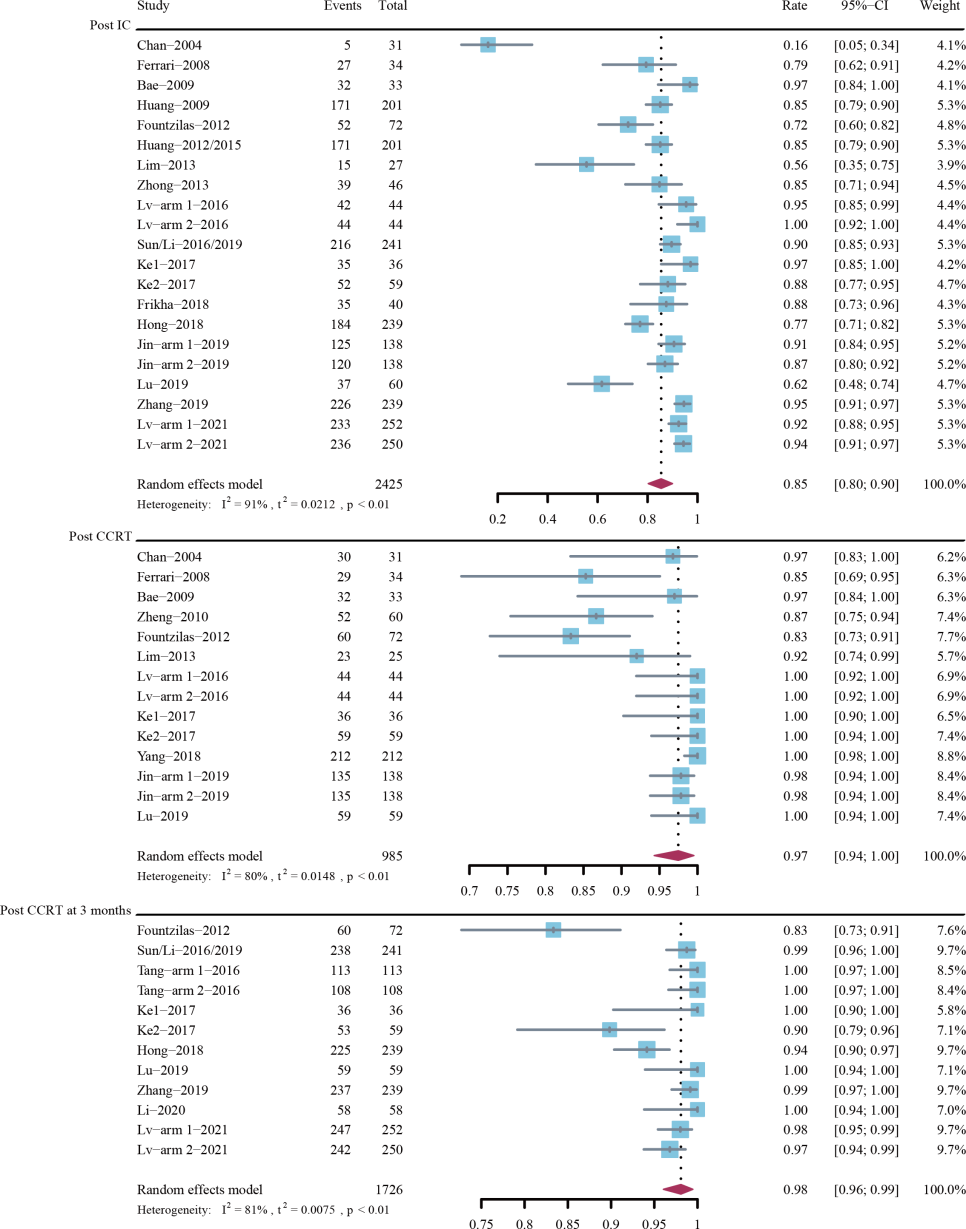
Objective response rate (ORR) post-induction chemotherapy (IC), post-concurrent chemoradiotherapy (CCRT), and post-CCRT at 3 months.

**Figure 3** depicts the forest plots for ORR. The estimated ORRs post induction chemotherapy, post CCRT, and post CCRT at 3 months were 85% (95% CI, 80%-90%; *I* ^2^ = 91%; P < 0.01), 97% (95% CI, 94%-100%; *I* ^2^ = 80%; P < 0.01), and 98% (95% CI, 96%-99%; *I* ^2^ = 81%; P < 0.01), respectively.

**Figure 4:**
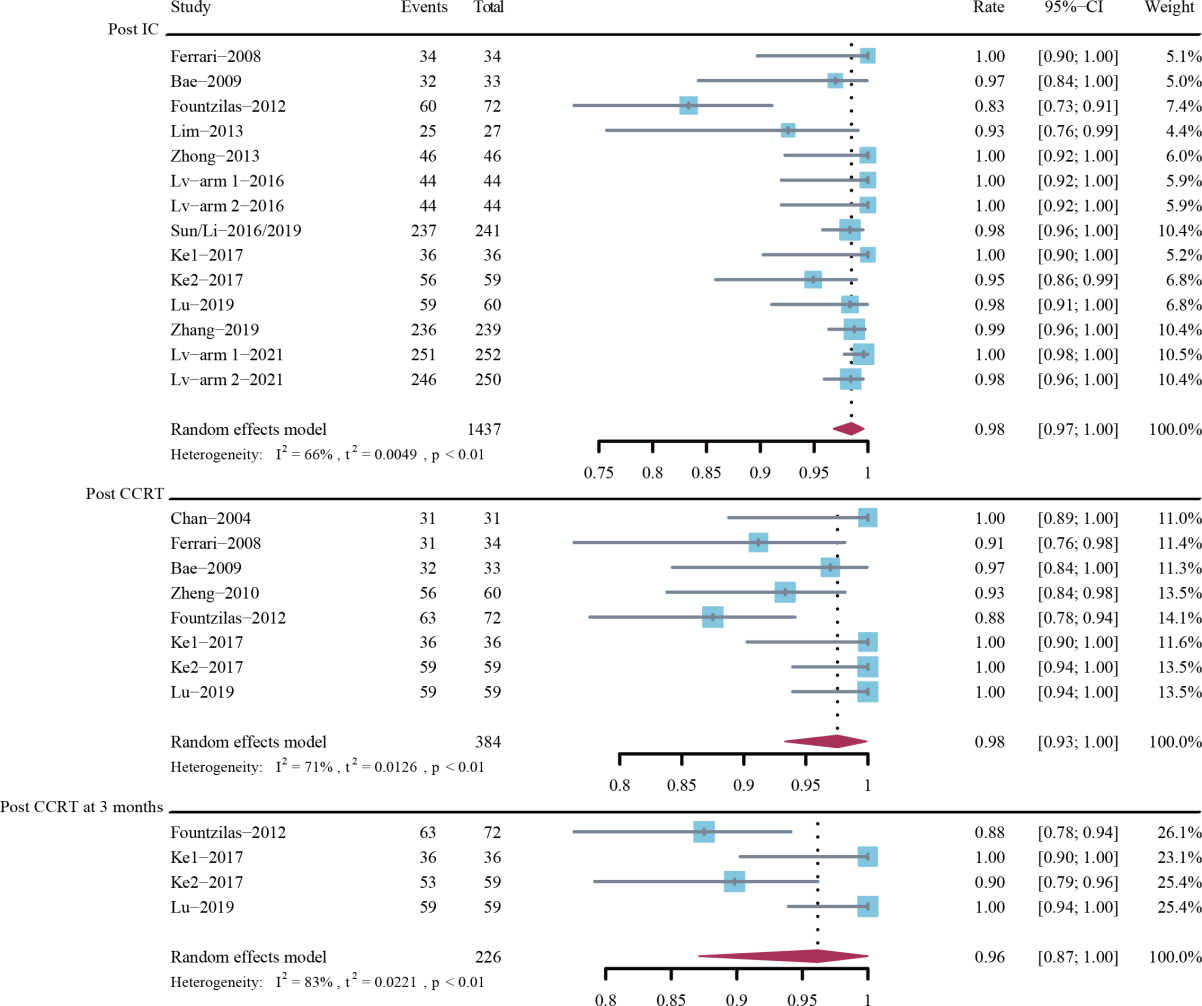
Disease control rate (DCR) post-induction chemotherapy (IC), post-concurrent chemoradiotherapy (CCRT), and post-CCRT at 3 months.

**Figure 4** depicts the forest plots for DCR. The estimated DCRs post induction chemotherapy, post CCRT, and post CCRT at 3 months were 98% (95% CI, 97%-100%; *I* ^2^ = 66%; P < 0.01), 98% (95% CI, 93%-100%; I^2^ = 71%; P < 0.01), and 96% (95% CI, 87%-100%; *I* ^2^ = 83%; P < 0.01), respectively.

### Subgroup analysis of survival outcomes and responses

**Figure 5:**
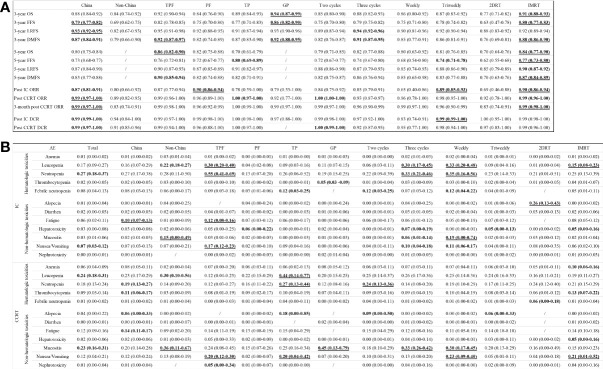
Subgroup analyses of survival outcomes, responses **(A)**, and adverse events **(B)** in terms of populations, regimens, cycles, and concurrent platinum strategies.

**Figure 5A** displays the subgroup analyses regarding population, induction chemotherapeutic regimens, induction chemotherapy cycles, and platinum-based CCRT strategies.

Patients in China achieved higher 3-year FFS (79% [95% CI, 77%-82%] vs. 69% [95% CI, 67%-75]) and LRFS (93% [92%-95%] vs. 82% [95% CI, 67%-93%]) rates, and ORRs (post CCRT: 99% [95% CI, 97%-100%] vs. 89% [95% CI, 82%-95%]; 3-month post CCRT: 99% [95% CI, 97%-100%] vs. 83% [95% CI, 74%-91%]) versus patients outside Chinese region.

GP induction chemotherapy was associated with the highest 3-year OS and FFS rates (OS: 94% [95% CI, 87%-99%]; FFS: 86% [82%-90%]), followed by TPF (92% [95% CI, 90%-94%]; 82% [78%-85%]), TP (89% [95% CI, 84%-93%]; 77% [71%-83%]), and PF (84% [95% CI, 76%-90%]; 75% [70%-80%]). In regard of 5-year OS with an absence of GP data, TPF was associated with the highest rate (86%; 95% CI, 82%-90%), followed by PF (82%; 95% CI, 75%-88%) and TP (70%; 95% CI, 61%-79%). In addition, PF (90%; 95% CI, 86%-94%) had a higher ORR after induction chemotherapy compared to TPF (87%; 95% CI, 77%-94%), GP (79%; 95% CI, 33%-100%), and TP (78%; 95% CI, 39%-100%).

In comparison with two cycles of induction chemotherapy, three cycles of induction chemotherapy might slightly increase the 3-year LRFS (94% [95% CI, 92%-96%] vs. 89% [95% CI, 83%-94%]) and DMFS (91% [95% CI, 87%-95%] vs. 82% [95% CI, 76%-87%]) rates, but fail to improve the ORR (93% [95% CI, 87%-97%] vs. 100% [95% CI, 100%-100%]) and DCR (92% [95% CI, 87%-95%] vs. 100% [95% CI, 99%-100%]) after the completion of CCRT.

Before the administration of platinum-based CCRT, patients who had received induction chemotherapy in the triweekly concurrent platinum therapy group had an 89% (95% CI, 85%-93%) of ORR and a 99% (95% CI, 99%-100%) of DCR that were much higher than the weekly group (65% [95% CI, 40%-86%]; 83% [95% CI, 74%-91%]). In addition, the triweekly group showed an increased 5-year FFS rate versus the weekly group (74% [95% CI, 71%-78%] vs. 68% [95% CI, 54%-80%]). However, patients in both groups achieved comparable rates of 3-year (87% [95% CI, 83%-92%] vs. 86% [95% CI, 80%-92%]) and 5-year (81% [95% CI, 76%-85%] vs. 80% [95% CI, 63%-92%]) OS.

Intensity-modulated radiotherapy (IMRT) has changed outcome of NPC patients significantly. Since three-dimensional radiotherapy (3DRT) data failed to separate from published trials, pooled IMRT and two-dimensional radiotherapy (2DRT) results were sub-analyzed. The IMRT group showed higher rates of post CCRT objective response at 3 months (99% [95% CI, 98%-100%] vs. 83% [95% CI, 74%-91%]), 5-year OS (84% [95% CI, 77%-90%] vs. 70% [95% CI, 64%-76%]), and 5-year PFS (77% [95% CI, 73%-80%] vs. 62% [95% CI, 55%-68%]). Additionally, IMRT could decrease the rate of distant metastasis compared with 2DRT (5-year DMFS: 87% [95% CI, 84%-89%] vs. 70% [95% CI, 63%-76%]).

### Incidences of grade 3 or higher adverse events and subgroup analysis

For the meta-analysis, we focused on the hematological and non-hematological grade 3 or higher adverse events that were recorded during the induction chemotherapy and CCRT phases. A comprehensive list of the incidences of anemia, leucopenia, neutropenia, thrombocytopenia, febrile neutropenia, alopecia, diarrhea, fatigue, hepatotoxicity, mucositis, nausea/vomiting, and nephrotoxicity is provided in **Figure 5B**.

During the induction chemotherapy phase, the most common hematological grade 3 or higher adverse events were neutropenia (27%; 95% CI, 18%-37%), leucopenia (17%; 95% CI, 9%-27%), and febrile neutropenia (8%; 95% CI, 4%-13%). The most common non-hematological grade 3 or higher adverse events were nausea/vomiting (7%; 95% CI, 3%-12%) and fatigue (6%; 95% CI, 2%-11%). Patients received TPF experienced the highest incidences of grade 3 or higher neutropenia (55%; 95% CI, 41%-69%), leucopenia (30%; 95% CI, 20%-40%), fatigue (12%; 95% CI, 8%-16%), and nausea/vomiting (17%; 95% CI, 12%-23%). Three cycles of induction chemotherapy induced more incidences of grade 3 or higher neutropenia (33% [95% CI, 21%-46%] vs. 22% [95% CI, 9%-39%]) and leucopenia (30% [95% CI, 17%-45%] vs. 6% [95% CI, 3%-11%]) against the two cycles group.

During the CCRT phase, the most common hematological grade 3 or higher adverse events were leucopenia (24%; 95% CI, 18%-31%), neutropenia (18%; 95% CI, 13%-24%), and thrombocytopenia (9%; 95% CI, 5%-14%). The most common non-hematological grade 3 or higher adverse events were mucositis (23%; 95% CI, 16%-31%), fatigue (12%; 95% CI, 9%-16%), and nausea/vomiting (12%; 95% CI, 4%-21%). Patients received TP induction chemotherapy had the highest incidences of grade 3 or higher leucopenia (44%; 95% CI, 14%-77%), neutropenia (27%; 95% CI, 13%-44%), mucositis (20%; 95% CI, 4%-42%), and alopecia (18%; 95% CI, 0%-85%). More cases of grade 3 or higher neutropenia (24% [95% CI, 13%-36%] vs. 14% [95% CI, 8%-20%]) were reported in the two cycles group, while more cases of grade 3 or higher mucositis (33% [95% CI, 26%-42%] vs. 18% [95% CI, 10%-29%]) were reported in the three cycles group. Additionally, patients treated with weekly platinum-based CCRT experienced higher incidences of grade 3 or higher mucositis (30% [95% CI, 17%-45%] vs. 20% [95% CI, 13%-29%]) and nausea/vomiting (23% [95% CI, 9%-40%] vs. 5% [95% CI, 1%-11%]) compared to the patients in the triweekly group. In terms of radiation techniques, patients in the IMRT group showed higher incidences of grade 3 or higher leucopenia (15% [95% CI, 8%-23%] vs. 1% [95% CI, 0%-4%]) and nausea/vomiting (21% [95% CI, 1%-52%] vs. 4% [95% CI, 0%-18%]) against the 2DRT group.

## Discussion

We performed a systematic review of induction chemotherapies and integrated the survival outcomes, responses, and toxic effects in patients with locoregionally advanced NPC who received induction chemotherapy and platinum-based CCRT. To our knowledge, this is the most comprehensive and largest meta-analysis of induction chemotherapy in NPC. Previous meta-analyses mainly demonstrated the benefits of adding induction chemotherapy to CCRT (9). Nevertheless, different populations, induction chemotherapeutic regimens and cycles, and even CCRT strategies may impact the efficacy and tolerability. A comprehensive analysis of the induction chemotherapeutic strategies reported in prospective clinical trials is essential, as the pooled data constitute a critical reference for clinicians. Significant heterogeneity existed among the enrolled studies, however, sensitivity analyses indicated that no substantial changes were found in the pooled survival outcomes and responses.

Although platinum-based induction chemotherapy significantly prolongs survival outcomes, whether adding 5-fluorouracil to TP provides more benefits is hard to judge. Up to now, several studies have compared the efficacy and safety data between TPF and TP. Xiong et al. indicated that TPF failed to improve the OS and PFS in stage III-IV NPC patients compared with TP (53). A Bayesian network meta-analysis of prospective clinical trials involving 1570 patients found that TPF had the highest probability of being the optimal regimen versus TP and PF in terms of OS (50% vs. 47% vs. 2%) (54). In our analysis, we noticed that patients in both TP and TPF subgroups achieved nearly 100% of ORR after completing induction chemotherapy and CCRT. However, TPF had much higher 5-year OS (86% vs. 70%) and DMFS (90% vs. 82%) rates against TP. These results were consistent with the retrospective study published by Tao et al. that patients received TPF had better 5-year OS (85% vs. 79%; *p* = 0.037), PFS (85% vs. 77%; *p* = 0.008) and DMFS (90% vs. 82%; *p* = 0.004) rates than patients received TP (55).

The integrated 3-year survival rates of GP in our analysis showed satisfying effects in treating NPC patients, including 3-year OS, FFS, and DMFS rates. In compared with TPF, GP showed a lower ORR after induction chemotherapy (79% vs. 87%) and comparable 3-year OS (94% vs. 92%), FFS (86% vs. 82%), LRFS (93% vs. 95%), and DMFS (92% vs. 92%) rates. In a comparative retrospective study, GP had a similar 3-year OS (94% vs. 92%), FFS (83% vs. 82%), LRFS (94% vs. 95%), and DMFS (90% vs. 90%) rates versus TPF, and no significant differences were observed (56). Nevertheless, GP induction chemotherapy was demonstrated to be cost-effective compared with TPF for locoregionally advanced NPC patients in real-world practice (57, 58).

On the other hand, published data have demonstrated that TPF achieved significantly better 10-year OS than PF (HR, 0.58; *p* = 0.005), and the difference between TP and PF was marginally significant (HR, 0.71; *p* = 0.056) (59). Regarding the 5-year data, TPF regimen significantly improved OS (88% vs. 81; *p* = 0.042) rate compared with the PF regimen (60). However, according to our analysis, PF had a better 5-year OS rate than TP (82% vs. 70%) and showed the highest ORR after induction chemotherapy (90%), followed by TPF (87%), GP (79%), and TP (78%). It seems hard to deduce that PF is the lowest effective induction chemotherapeutic regimen.

Anthracycline-based induction chemotherapeutic regimens include epirubicin + paclitaxel + cisplatin, epirubicin + cisplatin, and epirubicin + mitomycin C + cisplatin + 5-fluorouracil. These strategies were mainly applied in the non-China population and Taiwan participants (21, 27, 41). In Fountzilas’s study, locoregionally advanced NPC patients treated with epirubicin plus paclitaxel plus cisplatin had a 72% of ORR post-induction chemotherapy and an 83% of ORR post-CCRT (21). In Hong’s report, the ORR after induction chemotherapy was 78% (41). In comparison with our pooled data, the addition of epirubicin to TP may not critically improve the responses in NPC patients. Moreover, since the unreversible cardiotoxicity, epirubicin has a 900 mg/m2 of maximum cumulative dose.

For CCRT strategies, triweekly platinum-based CCRT showed a higher 5-year FFS versus the weekly group (74% vs. 68%) in our analysis, but OS results were similar. A previously pooled analysis of retrospective studies showed no significant differences in 5-year survival outcomes between weekly and triweekly treatments (61). However, the weekly strategy may be associated with improved quality of life than the triweekly regimen (62).

The addition of induction chemotherapy to CCRT has revolutionized the treatment of locoregionally advanced NPC, but the efficacy deserves further elevated. Regardless of complete clinical remission is attained after induction chemotherapy and CCRT, patients may suffer a high risk of locoregional relapse or distant metastasis. Chen et al. reported a phase 3 clinical trial in 2021 and indicated that adding metronomic adjuvant capecitabine after CCRT significantly improved survival outcomes with a manageable safety profile (63). In the subgroup analysis of Chen’s study, we noticed that only patients who received induction chemotherapy could benefit from adjuvant capecitabine treatment (FFS HR 0.49; 95% CI, 0.29-0.83) instead of patients who were treated with CCRT alone (FFS HR 0.51; 95% CI, 0.20-1.30). However, not all locoregionally advanced NPC patients are the suitable population for adjuvant chemotherapy. The changes of plasma EBV DNA across induction chemotherapy and CCRT may provide the necessity of the administration of adjuvant chemotherapy (64, 65). Finding out the suitable populations for induction chemotherapy plus CCRT, CCRT alone, and induction chemotherapy plus CCRT followed by adjuvant chemotherapy is meaningful for developing the treatment of NPC.

In terms of grade 3 or higher treatment-related adverse events, patients who received TPF regimen may suffer more incidences of leucopenia (30%), neutropenia (55%), fatigue (12%), and nausea/vomiting (17%) during the induction chemotherapy phase. In addition, three cycles of induction chemotherapy could induce more grade 3 or higher leucopenia (30%) and neutropenia (33%) versus two cycles. However, these toxicities are manageable. Thus, timely granulocyte colony-stimulating factor treatment could effectively prevent treatment-related severe adverse events or deaths.

## Strengths and limitations

The strengths of this analysis included (1) the results are supported by the large sample size from both single-arm and multi-arm prospective clinical trials, and (2) detailed subgroup analyses according to different populations, induction chemotherapeutic regimens, cycles, and CCRT strategies are displayed, because previously published meta-analyses mainly focused on the hazard ratios, odds ratios, or risk ratios in randomized studies comparing induction chemotherapy plus CCRT with CCRT alone or CCRT plus adjuvant chemotherapy. Nevertheless, our study has several limitations. First, heterogeneities existed among the enrolled studies. However, the large heterogeneity could mean that different clinical trials might exhibit inconsistent data of induction chemotherapy in treating locoregionally advanced NPC patients, which was the main point for us to conduct this meta-analysis to analyze the published data of induction chemotherapy comprehensively. In addition, a random-effects model was adopted all through this study to address the heterogeneity. Second, patients were treated with different cycles of induction chemotherapy. The primary reason for the discontinuation of induction chemotherapy was the adverse events, but most of the enrolled patients received two to three treatment cycles. Fortunately, the two-cycle group was not inferior to the three-cycle group. Third, in the CCRT phase, concurrent chemotherapies comprised weekly and triweekly strategies. Although heterogeneities may increase accordingly, our subgroup analysis and previously published pooled analysis had indicated no significant differences between weekly and triweekly strategies.

## Conclusions

This meta-analysis has defined survival outcomes, response rates, and the incidences of treatment-related adverse events in locoregionally advanced NPC patients who received induction chemotherapy followed by CCRT. Different population and induction regimens may be associated with different survivals, responses, and adverse events. This global overview of the effects and risks of induction chemotherapies can provide a reference for clinicians and may guide clinical practice for patients with locoregionally advanced NPC.

## Data availability statement

The original contributions presented in the study are included in the article/**Supplementary material**. Further inquiries can be directed to the corresponding author.

## Author contributions

B-CW and G-HL had full access to all of the data in the study and take responsibility for the integrity of the data and the accuracy of the data analysis. Concept and design, B-CW and G-HL. Acquisition, analysis, or interpretation of data: all authors. Drafting of the manuscript, B-CW, B-HK, and G-HL. Critical revision of the manuscript for important intellectual content, B-CW, X-XL, and QL. Statistical analysis, B-CW and G-HL. Administrative, technical, or material support, QL. Supervision, QL. All authors contributed to the article and approved the submitted version.

## Funding

This study was supported by the Hubei Provincial Natural Science Foundation (Grant number: 2020CFB397 to B-CW) and the Independent Innovation Foundation of Wuhan Union Hospital (Grant number: 2019-109 to B-CW).

## Conflict of Interest

The authors declare that the research was conducted in the absence of any commercial or financial relationships that could be construed as a potential conflict of interest.

## Publisher’s note

All claims expressed in this article are solely those of the authors and do not necessarily represent those of their affiliated organizations, or those of the publisher, the editors and the reviewers. Any product that may be evaluated in this article, or claim that may be made by its manufacturer, is not guaranteed or endorsed by the publisher.
