# Supplementary material for: Induction chemotherapy in locoregionally advanced nasopharyngeal carcinoma: A systematic review and meta-analysis
Source: Front Oncol. 2022 Jul 29;12:927510. doi: 10.3389/fonc.2022.927510 (PMC9373136; doi:10.3389/fonc.2022.927510)

**Supplement 1**

**eFigure 1** Flow Chart of the Study Selection Process. (Irrelevant record included other type of tumor studies, metastatic diseases, non-induction chemotherapy strategies, and other irrelevant topics.)


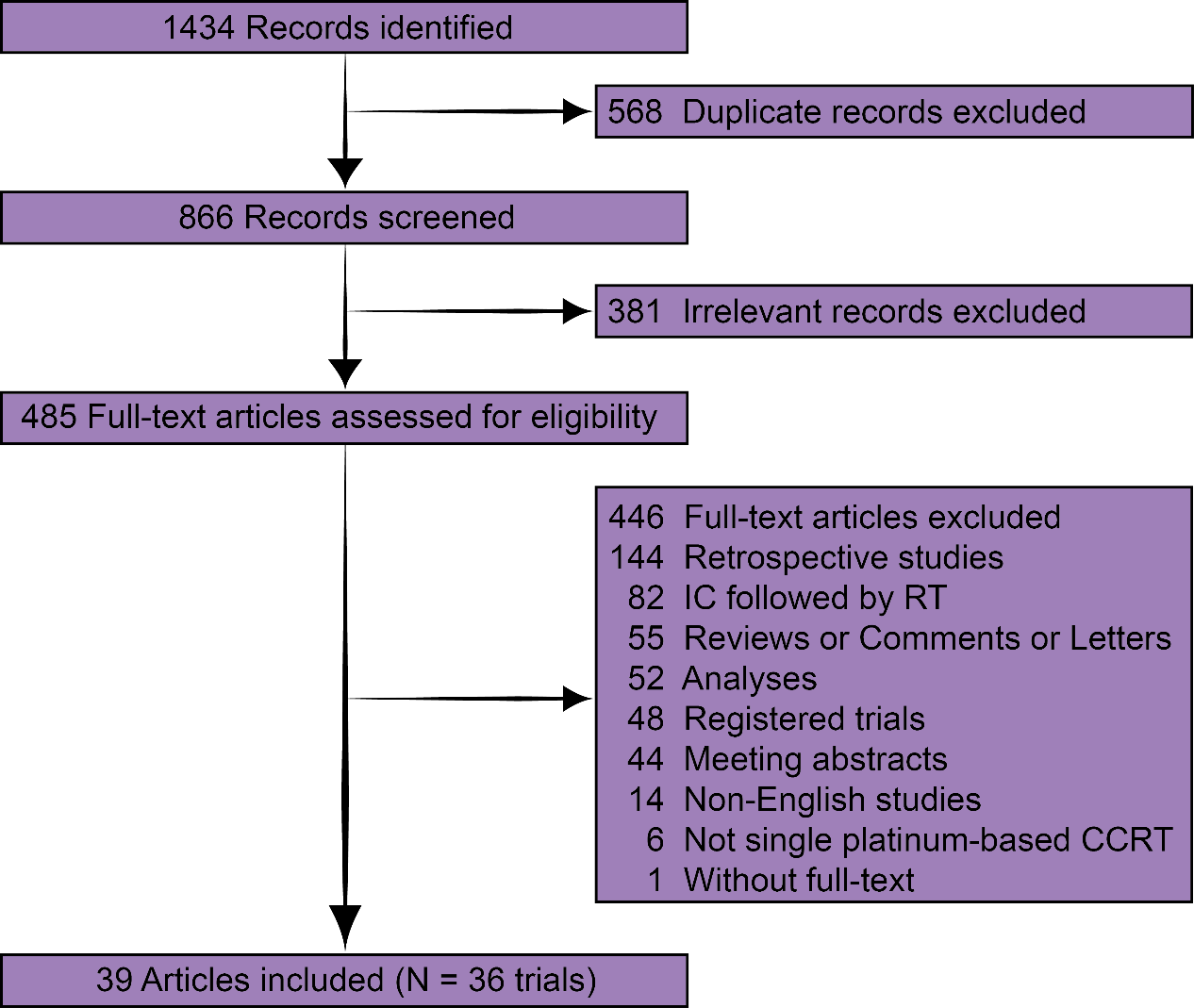

Supplement: Supplementary file 1 [file DataSheet_1.docx]
