# Supplementary material for: Induction chemotherapy in locoregionally advanced nasopharyngeal carcinoma: A systematic review and meta-analysis
Source: Front Oncol. 2022 Jul 29;12:927510. doi: 10.3389/fonc.2022.927510 (PMC9373136; doi:10.3389/fonc.2022.927510)

**Supplement 2**

**eTable 1 Quality evaluation for each study enrolled.**

| Study | Jadad score |
| --- | --- |
| Chan-2004 | 1 |
| Ferrari-2008 | 1 |
| Bae-2009 | 1 |
| Huang-2009 | 3 |
| Hui-2009 | 3 |
| Kong*-2010 | 1 |
|  | 1 |
| Zheng-2010 | 1 |
| Fountzilas-2012 | 3 |
| Huang-2012/2015 | 3 |
| Kong*-2013 | 1 |
| Lim-2013 | 1 |
| Zhong-2013 | 1 |
| Rosenblatt-2014 | 3 |
| Lee-2015/2020 | 3 |
| Tan-2015 | 3 |
| Lv-2016 | 3 |
| Sun-2016  Li-2019 | 3 |
| Tang-2016 | 3 |
| Cao-2017  Yang-2019 | 3 |
| Ke-1-2017 | 1 |
| Ke-2-2017 | 1 |
| Kong*-2017 | 1 |
| Frikha-2018 | 3 |
| Hong-2018 | 3 |
| Wei-2018 | 1 |
| Yang-2018 | 3 |
| Ghosh-Laskar-2019 | 1 |
| Jin-2019 | 3 |
| Lu-2019 | 3 |
| Zhang-2019 | 3 |
| Zhao-2019 | 1 |
| Al-Rajhi-2020 | 3 |
| Li-2020 | 3 |
| Lv-2021 | 3 |
| Yao-2021 | 1 |

Abbreviation: *, included two trials

**eFigure 1** Funnel plots for 3-year overall survival (OS) (A), 3-year failure-free survival (FFS) (B), 3-year locoregional recurrence-free survival (LRFS) (C), 3-year distant metastasis-free survival (DMFS) (D), 5-year OS (E), 5-year FFS (F), 5-year LRFS (G), 5-year DMFS (H),


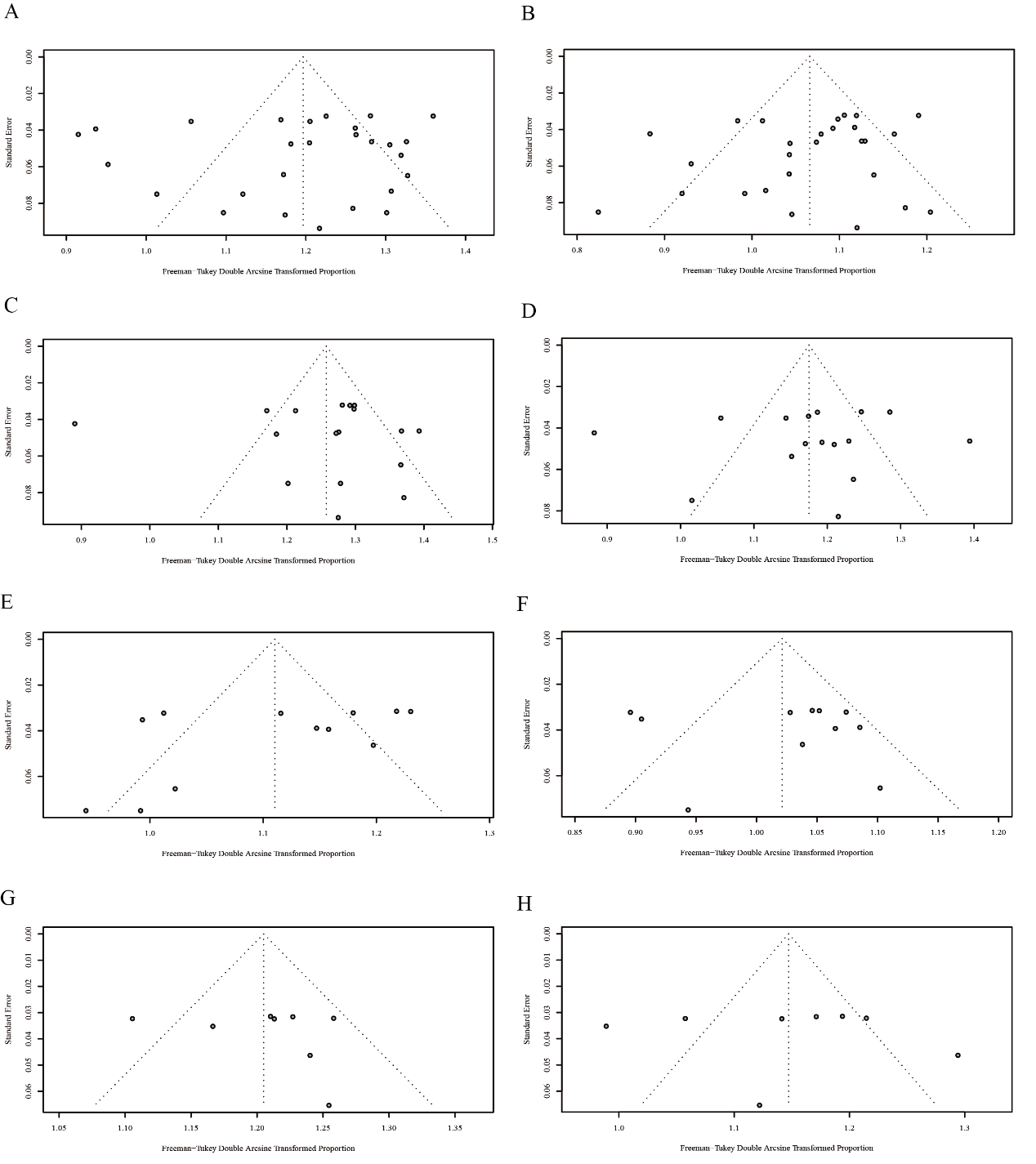


**eFigure 2** Funnel plots for objective response rate (ORR) post induction chemotherapy (IC) (A), post CCRT (B), and post CCRT at 3 months (C) and disease control rate (DCR) post induction chemotherapy (IC) (D), post CCRT (E), and post CCRT at 3 months (F).

**
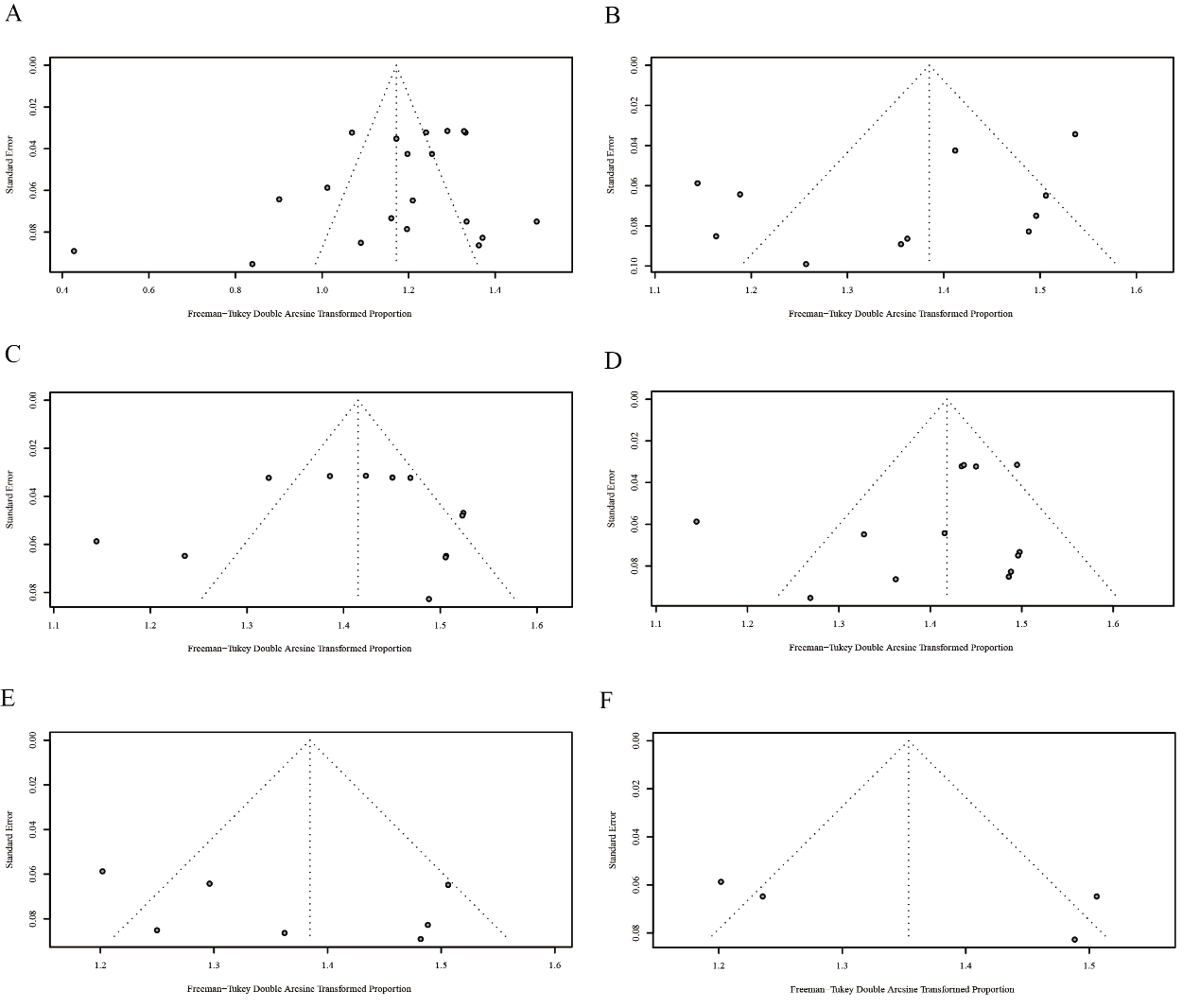
**

**eFigure 3** Egger’s tests for 3-year overall survival (OS) (A), 3-year failure-free survival (FFS) (B), 3-year locoregional recurrence-free survival (LRFS) (C), 3-year distant metastasis-free survival (DMFS) (D), 5-year OS (E), 5-year FFS (F), 5-year LRFS (G), 5-year DMFS (H).


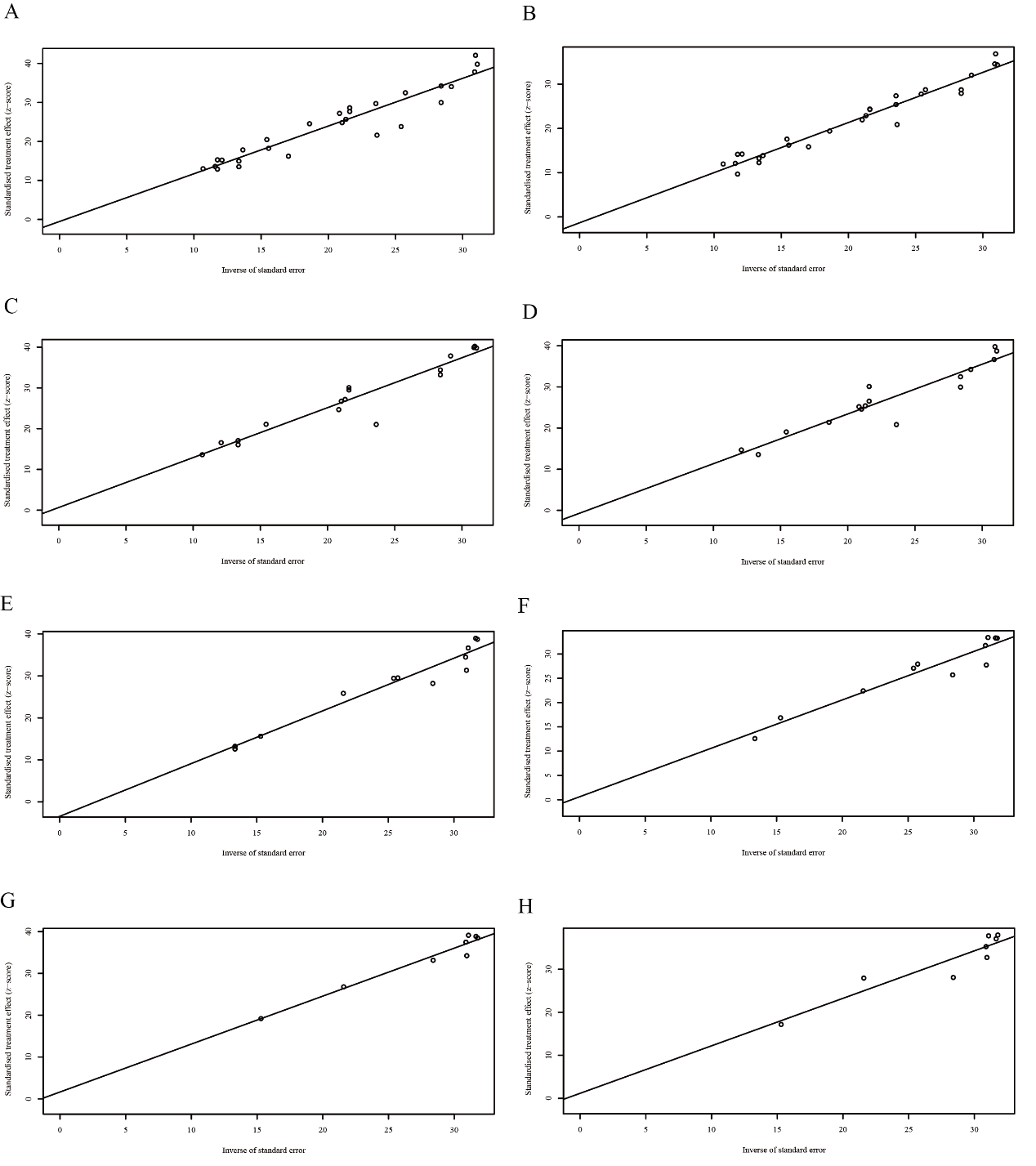


**eFigure 4** Egger’s tests for objective response rate (ORR) post induction chemotherapy (IC) (A), post CCRT (B), and post CCRT at 3 months (C) and disease control rate (DCR) post induction chemotherapy (IC) (D), post CCRT (E), and post CCRT at 3 months (F).


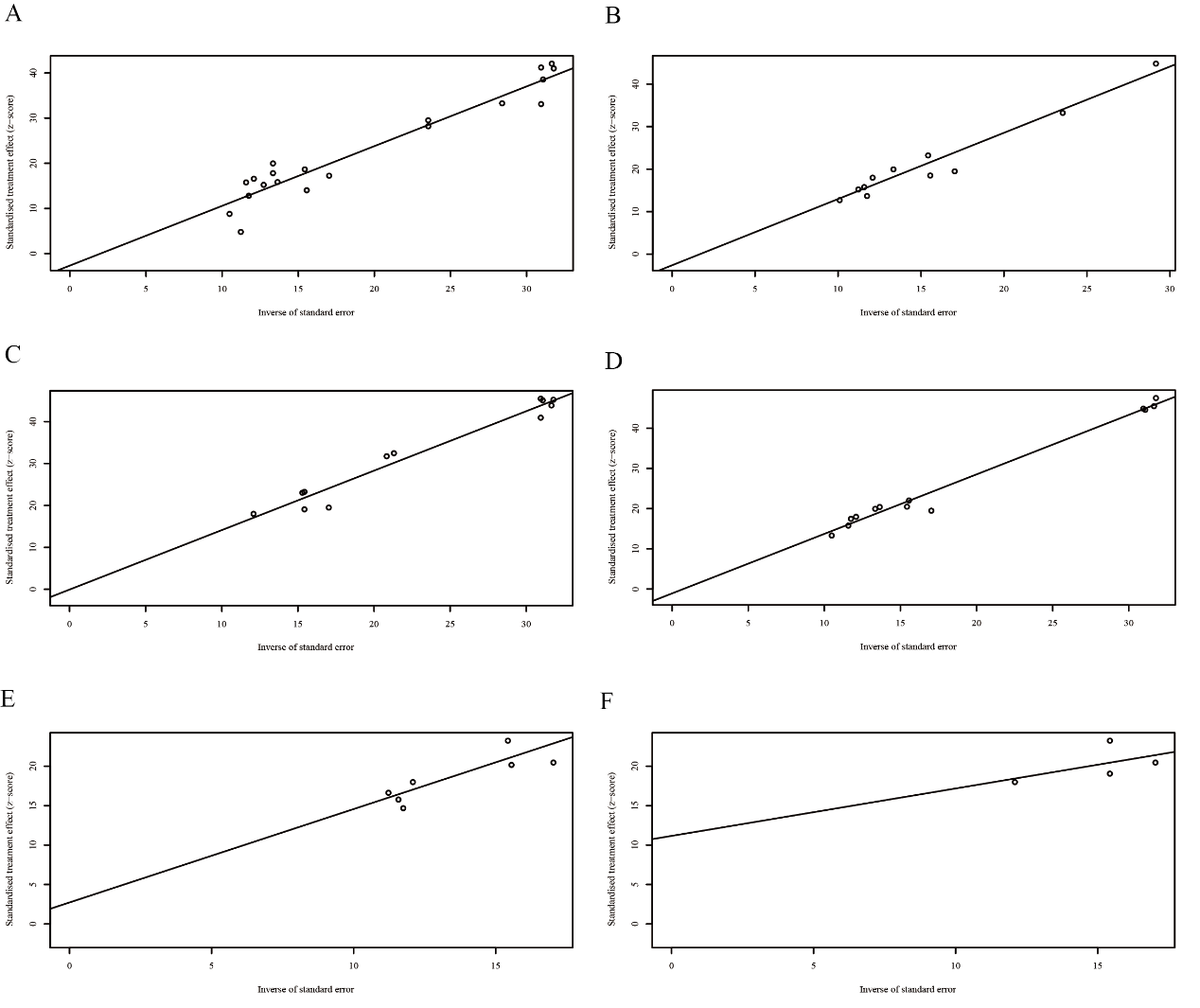


**eFigure 5** Begg’s tests for 3-year overall survival (OS) (A), 3-year failure-free survival (FFS) (B), 3-year locoregional recurrence-free survival (LRFS) (C), 3-year distant metastasis-free survival (DMFS) (D), 5-year OS (E), 5-year FFS (F), 5-year LRFS (G), 5-year DMFS (H).


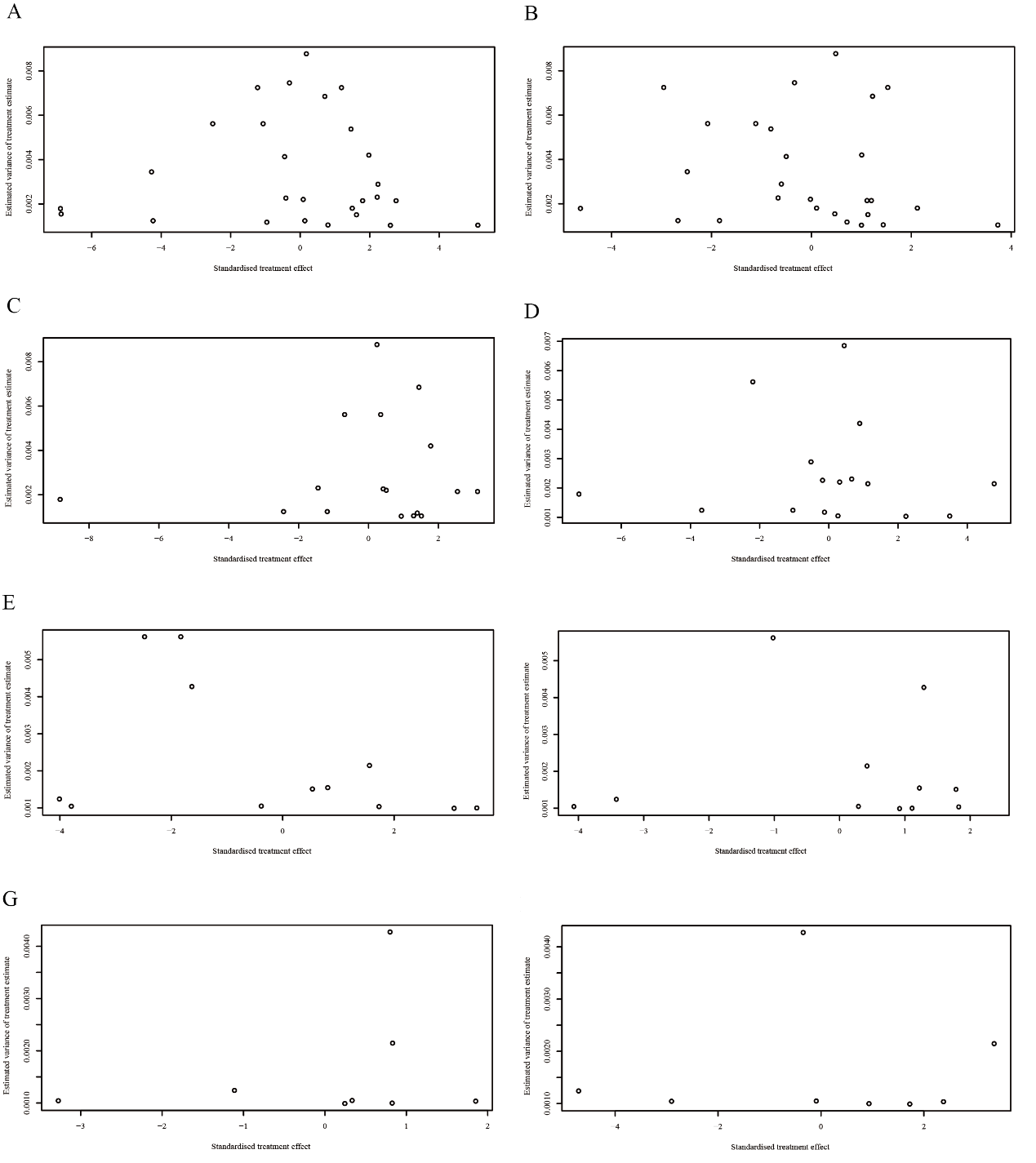


**eFigure 6** Begg’s tests for objective response rate (ORR) post induction chemotherapy (IC) (A), post CCRT (B), and post CCRT at 3 months (C) and disease control rate (DCR) post induction chemotherapy (IC) (D), post CCRT (E), and post CCRT at 3 months (F).

**
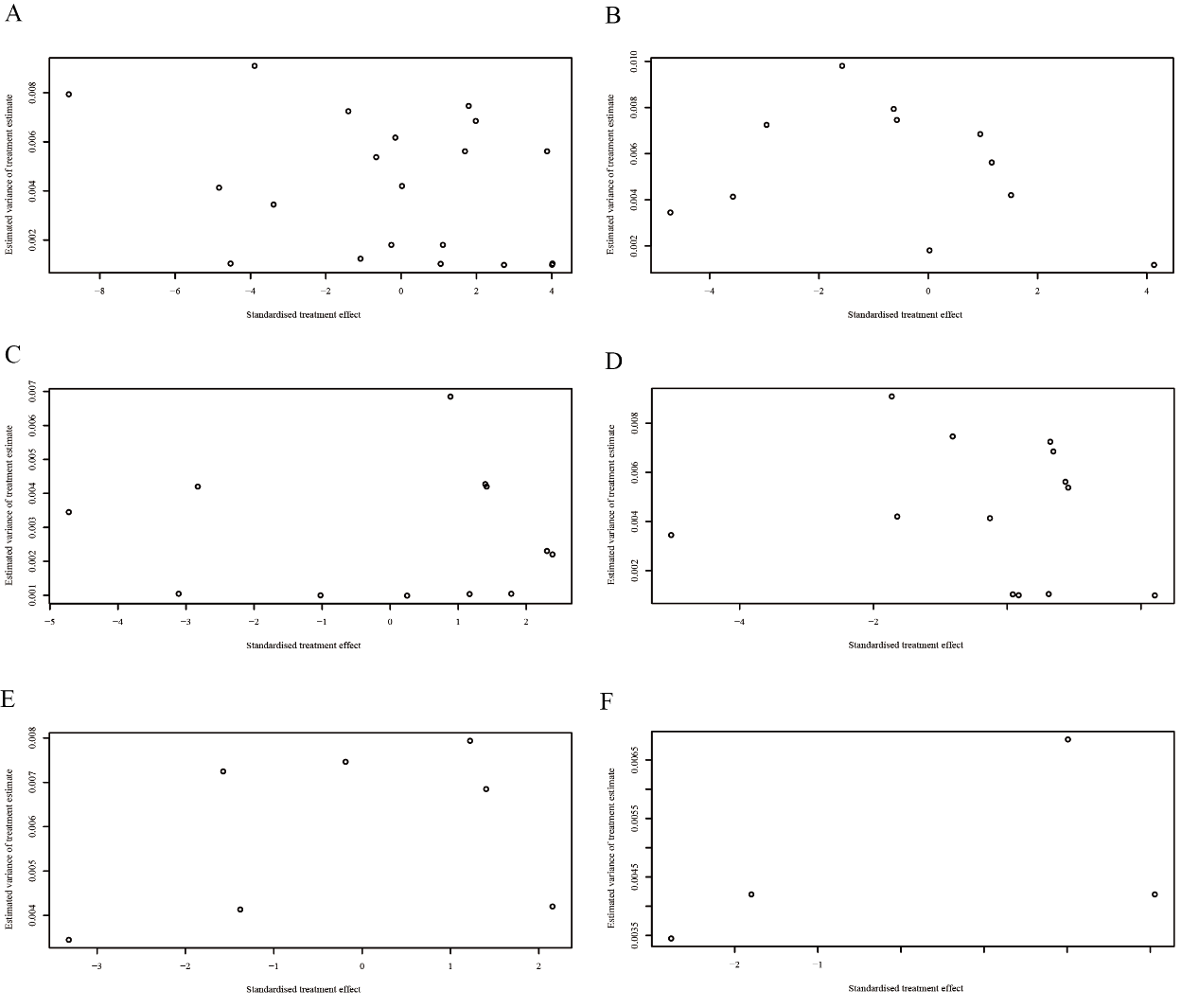
**

**eFigure 7** Sensitivity analysis of 3-year survival outcomes. (A) overall survival (OS); (B) 3-year failure-free survival (FFS); (C) 3-year locoregional recurrence-free survival (LRFS); (D) 3-year distant metastasis-free survival (DMFS).

**
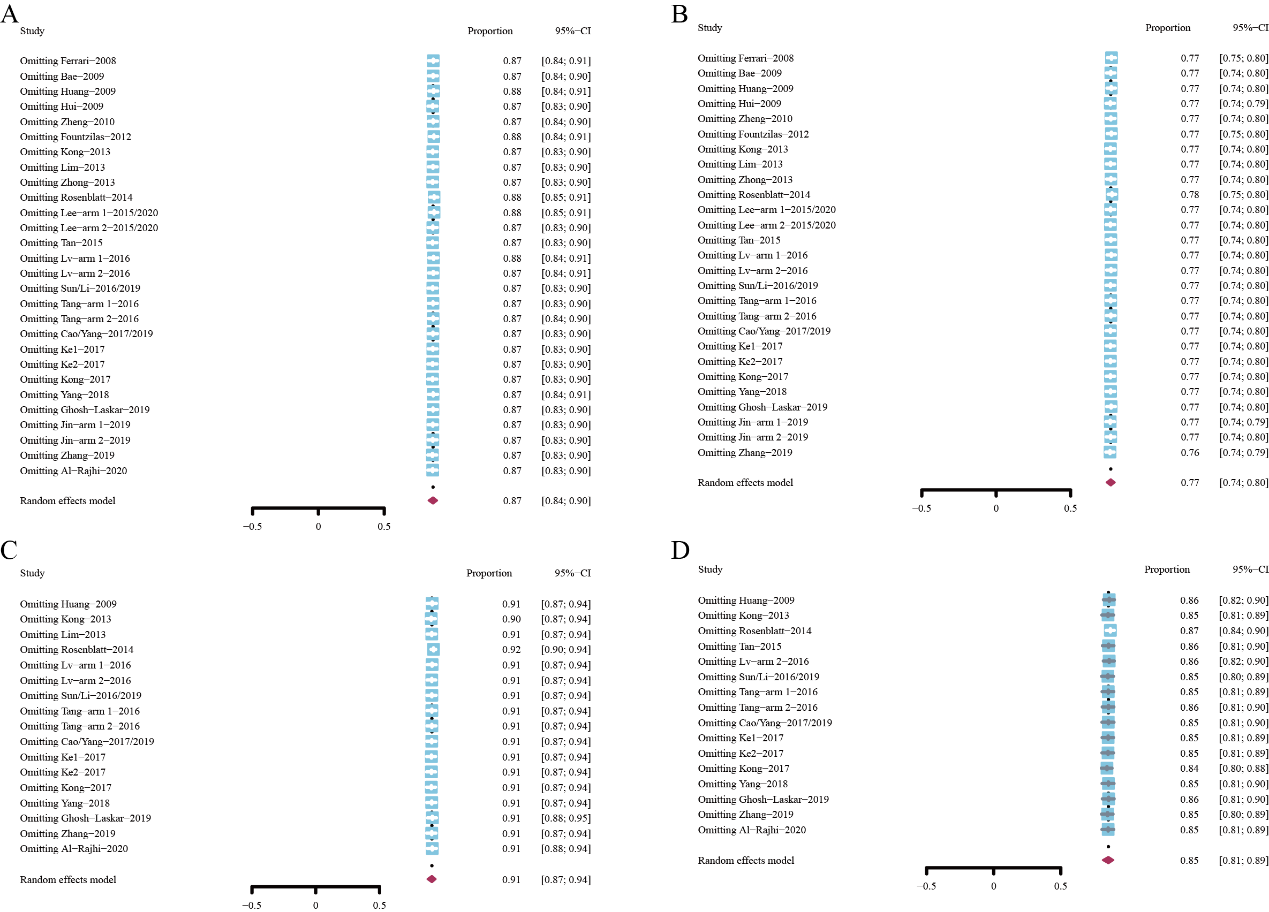
**

**eFigure 8** Sensitivity analysis of 5-year survival outcomes. (A) overall survival (OS); (B) 3-year failure-free survival (FFS); (C) 3-year locoregional recurrence-free survival (LRFS); (D) 3-year distant metastasis-free survival (DMFS).


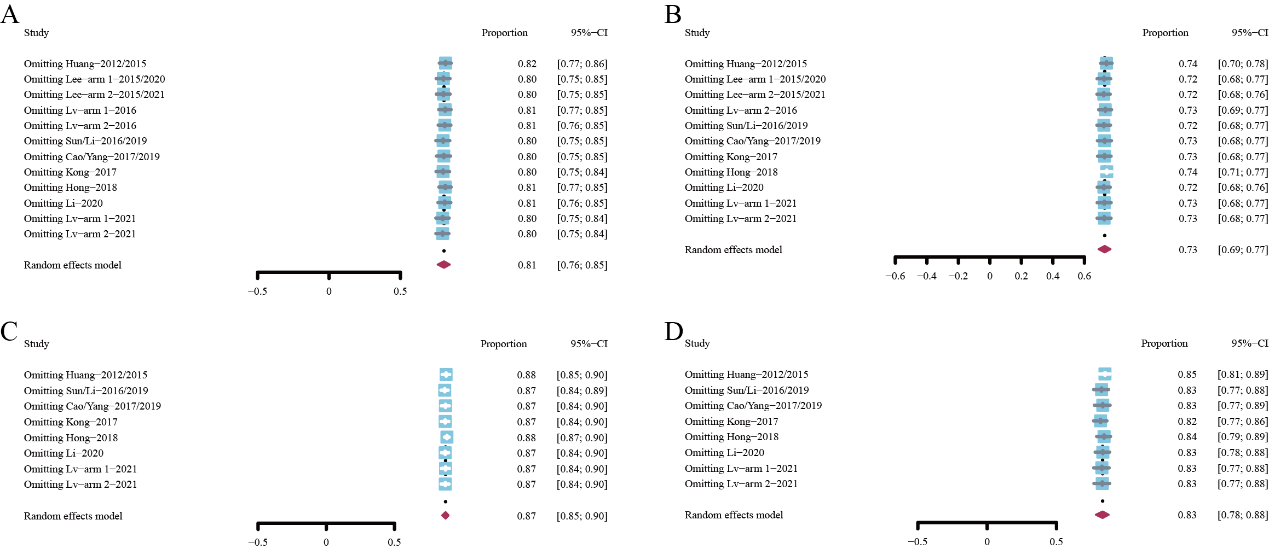


**eFigure 9** Sensitivity analysis of objective response rates. (A) post induction chemotherapy (IC); (B) post concurrent chemoradiotherapy (CCRT); (C) post CCRT at 3 months.

**
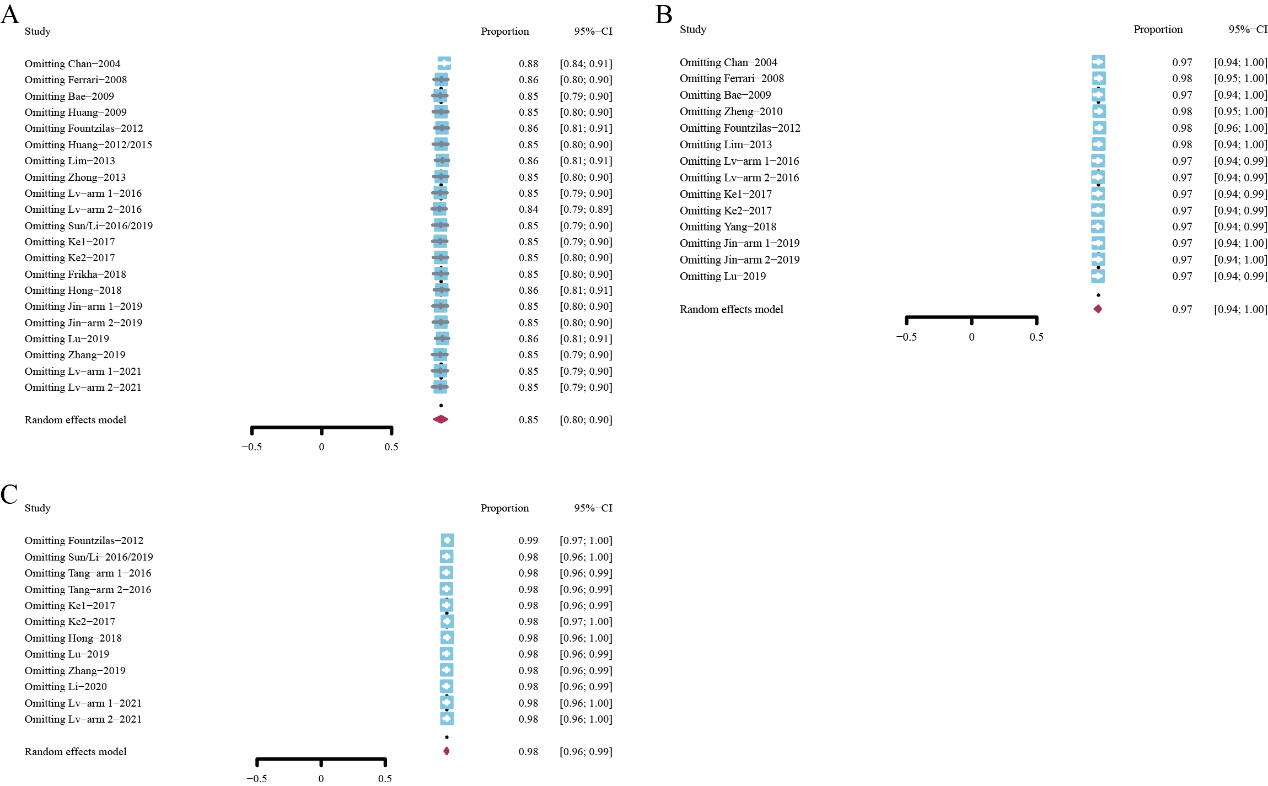
**

**eFigure 10** Sensitivity analysis of disease control rates. (A) post induction chemotherapy (IC); (B) post concurrent chemoradiotherapy (CCRT); (C) post CCRT at 3 months.


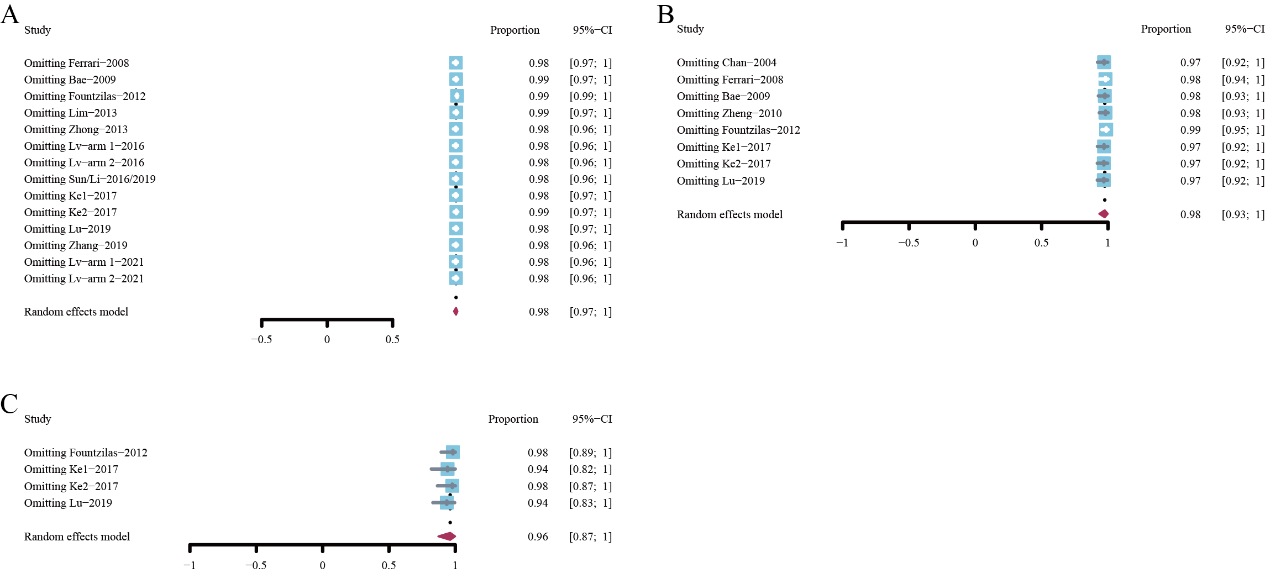

Supplement: Supplementary file 2 [file DataSheet_2.docx]
